# Prader-Willi Syndrome Presenting With Early Infantile Hypotonia: A Case Report

**DOI:** 10.7759/cureus.105369

**Published:** 2026-03-17

**Authors:** Aysha M Alsindi, Rehab G Amer, Lara M Boustros, Minoosh M Nasef

**Affiliations:** 1 Pediatrics, King Hamad University Hospital, Muharraq, BHR; 2 Neonatology, King Hamad University Hospital, Muharraq, BHR

**Keywords:** endocrine dysfunction, genetic mutation, hypotonia, prader-willi syndrome, sleep disorders

## Abstract

Prader-Willi syndrome (PWS) is a genetic disorder resulting from the loss of paternally expressed genes on chromosome 15q11.2-15q13.3. It is characterized by distinct clinical features and multisystem involvement, including endocrine, neurodevelopmental, and metabolic abnormalities. Early diagnosis can be challenging because clinical manifestations in infancy are often subtle; therefore, molecular testing is essential for confirmation. This report describes a one-year-old child newly diagnosed with PWS, aiming to highlight the clinical presentation and correlate the findings with current genetic and phenotypic evidence.

## Introduction

Prader-Willi syndrome (PWS) is a complex genetic condition caused by a deficiency of paternally expressed genes on chromosome 15 (15q11.3-15q13.3 region) [1]. The estimated incidence of PWS ranges between one in 16,000 and one in 21,000 live births [2]. Regarding etiology, the main genetic mechanisms that cause PWS are: a large deletion on the paternally inherited chromosome 15q11-15q13 region, seen in about 70% of cases; maternal uniparental disomy (UPD), which accounts for roughly 20-25% of cases; an abnormal imprinting defect, responsible for about 2-4% of cases; and less than 1% result from uncommon causes such as translocations and microdeletions. Importantly, paternally expressed genes in 15q11-15q13 are essential for hypothalamic development. This region contains protein-coding genes and noncoding RNAs, some of which are implicated in alternative splicing regulation and are abundantly expressed in the brain, acting primarily by modifying ribosomal RNAs [3]. Clinically, characteristic features include bifrontal narrowing, almond-shaped eyes, micrognathia with a high-arched palate, small hands and feet, as well as short stature resulting from multiple hormonal deficiencies affecting the endocrine, gonadal, pancreatic, adrenal, and thyroid systems [4].

In addition, neonatal features may include lethargy, poor feeding, thickened saliva, increased head-to-chest circumference ratio, and genital hypoplasia in both sexes, with cryptorchidism frequently observed in males [5]. During infancy, PWS is characterized by hypotonia, genital hypoplasia, respiratory issues, and feeding difficulties, progressing by the age of two years to developmental delay, learning disability, and hyperphagia due to impaired satiety [3]. Moreover, patients with PWS exhibit intellectual disability characterized by cognitive impairment, delayed motor and language milestones, learning difficulties, and deficits in social and emotional functioning [2].

With regard to diagnosis, PWS is challenging because the early clinical signs are often subtle during infancy, and the features become more recognizable later when hyperphagia and morbid obesity develop [1]. Nevertheless, clinicians often struggle to determine when to order molecular testing for classical PWS due to its overlapping features with syndromes such as Prader-Willi-like syndrome (PWLS) [3]. Therefore, this study presents a case of a one-year-old child recently diagnosed with PWS, with emphasis on the clinical picture in correlation with the literature.

## Case presentation

**Figure 1:**
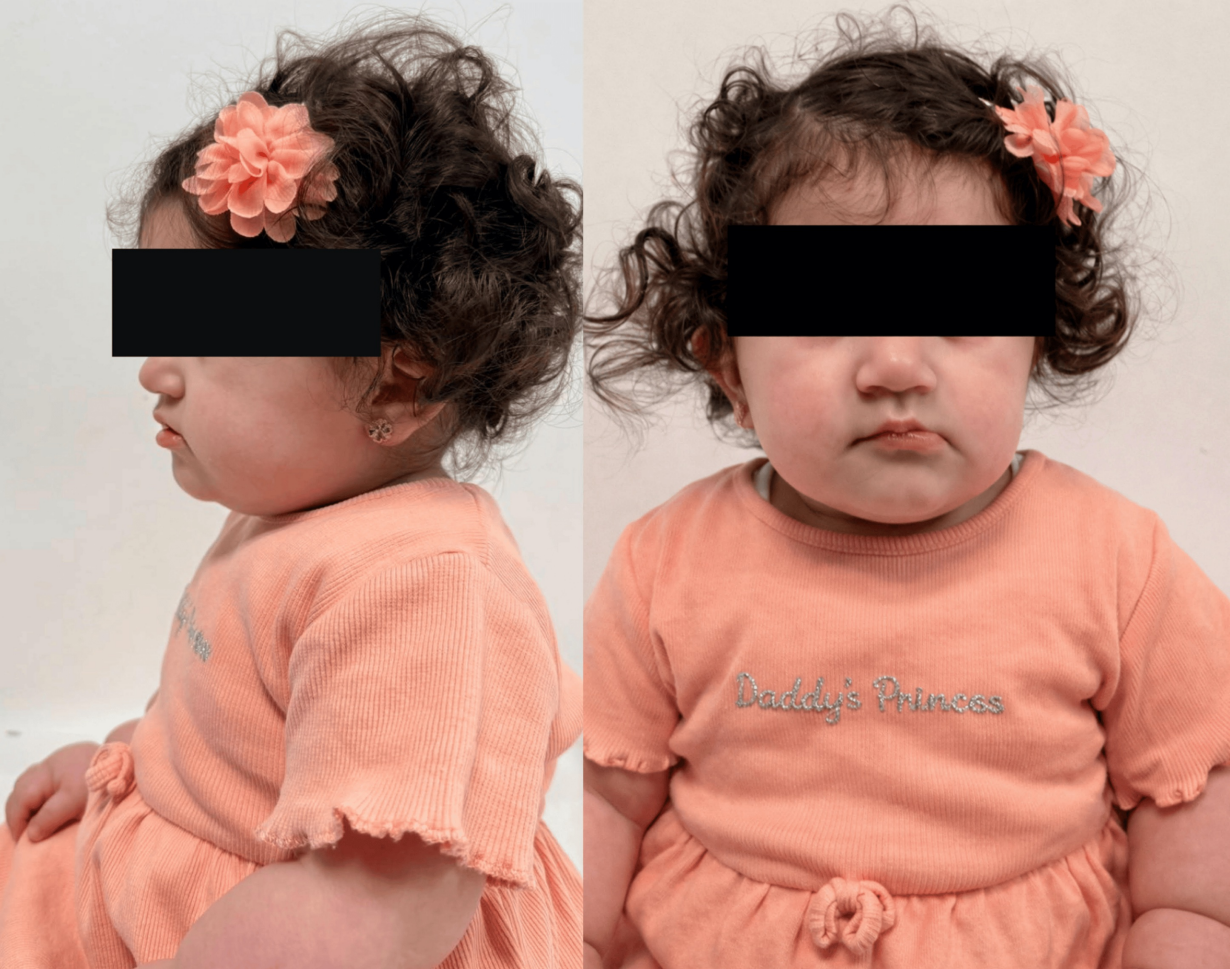
Facial features suggestive of PWS, including a round face, small mouth with a thin upper vermilion border, and downturned corners. PWS, Prader-Willi syndrome

**Figure 2:**
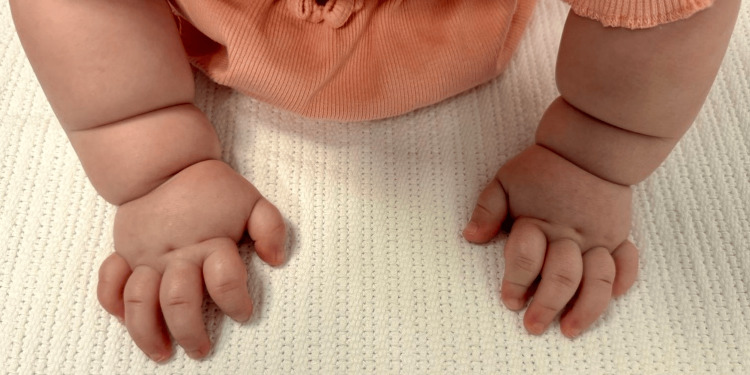
Patient with PWS showing small hands. PWS, Prader-Willi syndrome

The child was born at 37+1 weeks of gestation via category II cesarean section performed for failure of induction. Birth weight was 1.997 kg, with Apgar scores of eight and nine at one and five minutes, respectively, and a head circumference of 31 cm. She was admitted to the Neonatal Intensive Care Unit (NICU) for 20 days with an initial impression of intrauterine growth restriction (IUGR), low birth weight, and for further genetic evaluation, as the antenatal scan had suggested a chromosomal abnormality consistent with either Angelman syndrome (AS) or PWS. The patient demonstrated dysmorphic features characteristic of PWS, including a round facial appearance, almond-shaped eyes, deep-set eyes with narrow palpebral fissures, a broad nasal bridge, and bifrontal narrowing. Additional findings included a thin upper vermilion border with downturned corners of the mouth, small hands and feet, and short stature (Figure 1 and Figure 2).

During the NICU admission, the patient remained clinically stable. She required oxygen via nasal cannula for two hours after birth, after which she was weaned to room air without respiratory distress and progressively tolerated enteral feeds. Blood and urine cultures were obtained, and empirical intravenous antibiotics (ampicillin and amikacin) were initiated but subsequently discontinued following negative culture results. Notably, the infant exhibited hypotonia and poor feeding, although oral tolerance improved gradually.

Genetic testing was pursued, and fluorescence in situ hybridization (FISH) revealed a deletion of the SNRPN gene on chromosome 15q11.2, supporting the diagnosis of PWS/AS. Further molecular studies, including multiplex ligation-dependent probe amplification (MLPA), identified a heterozygous deletion in the MKRN3, MAGEL2, NDN, SNRPN, OCA2, GABRB3, ATP10A, and UBE3A genes within the 15q11 PWS/AS critical region. A reduced copy number ratio indicated a deletion consistent with PWS or AS. In addition, methylation-specific MLPA (MS-MLPA) demonstrated aberrant methylation in the SNRPN and MAGEL2 domains on 15q11. These are paternally inherited genes, and a copy number ratio of 0.5 with complete methylation of paternal alleles confirmed the diagnosis of PWS (Table 1 and Table 2).

**Table 1:** Pathogenic variant consistent with PWS. PWS, Prader-Willi syndrome

Si. no.	Deletions/duplications	No. of probes showing deletion/duplication/methylation	MLPA probe ratio (dosage quotient)	Disease	Classification
1	Heterozygous deletion	34	0.44-0.60	Prader-Willi syndrome	Pathogenic
2	Aberrant methylation	7	0.93-1.06		

**Table 2:** Reference showing interpretation of copy number and methylation status. In this row, the paternal and maternal copies of the 15q11 region are indicated with P for paternal and M for maternal, respectively. PWS, Prader-Willi syndrome; AS, Angelman syndrome

	PWS deletion	PWS disomy	Reference/wild type	AS disomy	AS deletion
Genomic situation of the 15q11 region*	M_	MM	PM	PP	P_
Copy number	1	2	2	2	1
Copy number ratio	0.5	1	1	1	0.5
% methylated	100%	100%	50%	0%	0%
Ratio after digestion	1	1	0.5	0	0

At one month of age, the patient was admitted to the pediatric ward following her first episode of an unprovoked seizure. The event was characterized by rapid, jerky movements of the upper limbs, upward eye deviation, and generalized stiffness lasting for a few seconds, followed by a postictal phase. The clinical impression was myoclonic seizure, and she was started on levetiracetam (Keppra) at 40 mg/kg/day. Initial electroencephalography (EEG) was normal; however, a repeat EEG during outpatient follow-up demonstrated abnormal left temporal sharp discharges. Brain MRI and echocardiography were performed and were unremarkable. Table 3 shows the latest laboratory investigations for the patient.

**Table 3:** Latest laboratory investigations for the patient. IGF-1, insulin-like growth factor 1; TSH, thyroid-stimulating hormone; FT4, free T4 thyroid hormone; ALPI, alkaline phosphatase; ALT, alanine aminotransferase; GGT, gamma-glutamyltransferase; AST, aspartate aminotransferase; HBA1C, hemoglobin A1C; VLDL, very-low-density lipoprotein cholesterol; LDL, low-density lipoprotein cholesterol; HDL, high-density lipoprotein cholesterol

Investigation	Result	Reference range
White blood cells	7.28x10^9^/L	5.0-16.0
Red blood cells	4.59x10^12^/L	3.0-5.4
Hemoglobin	11.9 g/dL	10.0-14.0
Hematocrit	37.1%	28.0-40.0
Mean corpuscular volume	80.8 fL	72.0-90.0
Mean corpuscular hemoglobin	26.0 pg	24.0-32.0
Red cell distribution width	14.4%	11.5-16.0
Platelets	305.0x10^9^/L	150.0-450.0
IGF-1	56.8 ng/mL	43.03-212.64
TSH	1.50 uIU/mL	0.35-5.5
FT4	1.21 ng/dL	0.73-2.33
Total protein	69 g/L	60.0-83.0
Globulin	20.80 g/L	20.0-35.0
Bilirubin total	3.6 umol/L	-
Bilirubin direct	<2.00 umol/L	0.0-5.0
ALP	356.0 U/L (high)	90.0-180.0
ALT	23.0 U/L	16.0-63.0
GGT	16 U/L	0.0-40.0
AST	53.0 U/L (high)	15.0-40.0
Cholesterol	5.7 mmol/L (high)	3.6-5.2
Triglycerides	1.58 mmol/L	0.0-1.7
VLDL cholesterol	0.31 mmol/L	0.1-1.7
LDL Cholesterol	4.69 mmol/L	1.6-4.7
HDL cholesterol	1.0 mmol/L	0.83-1.86
Total cholesterol/HDL ratio	5.7 (high)	0.0-5.0
HBA1C	5.5%	4.5-6.0
Anti-cytomegalovirus IgM	Non-reactive	
Anti-rubella IgM	Non-reactive	
Anti-Toxoplasma IgM	Non-reactive	

During subsequent follow-up with a pediatric neurologist, the levetiracetam dose was titrated upward to achieve seizure control. By seven months of age, she continued to demonstrate hypotonia, poor head control, and inability to roll over, although she was able to hear, visually fix and follow, grasp objects, and laugh. At 11 months of age, she was evaluated by pediatric endocrinology, and the importance of early initiation of growth hormone (GH) therapy was discussed with the family. GH therapy was considered essential for growth support, motor development, and cognitive outcomes in PWS. Prior to therapy initiation, she was referred to pediatric otolaryngology to exclude adenotonsillar hypertrophy and to assess for sleep-disordered breathing (SDB), as these are relative contraindications to GH therapy. Evaluation revealed no evidence of adenotonsillar hypertrophy or clinical signs suggestive of obstructive sleep apnea, and a sleep study was pending at the time of reporting.

At the follow-up visit at 13 months of age, the pediatric neurology assessment showed improvement in her developmental milestones. She demonstrated better muscle tone and head control and was able to roll both ways, although she was not yet crawling. She was able to hear, fix and follow, grasp objects, and exhibited appropriate social interaction. She laughed, vocalized “mama” and “dada,” understood the word “no,” and could wave bye-bye, clap, and dance.

## Discussion

Early diagnosis of PWS is critical, as it significantly improves long-term outcomes. Prenatal diagnosis can be established using molecular genetic testing of chorionic villi or amniotic fluid cells [1]. PWS is considered a contiguous gene syndrome resulting from the loss of at least two paternally expressed genes, including SNRPN, MKRN3, MAGEL2, and NDN, as well as several small nucleolar RNAs (snoRNAs). Although the specific genes responsible for the phenotype have not been fully delineated, their disruption is believed to play a central role in disease pathogenesis [3]. However, deletion of the SNORD116 gene appears to account for most clinical manifestations observed [6].

PWLS shares overlapping phenotypic characteristics, reflecting alterations in related molecular pathways [3]. One study reported weak fetal movement at 39 weeks of gestation, which has been associated with PWS [1]. Another study mentioned that characteristic prenatal hypotonia contributes to diminished fetal movements, malpresentation at delivery, and an increased frequency of assisted or cesarean births [6]. Postnatally, diagnosis can be established through a variety of genetic techniques, including high-resolution banding (HRB), FISH study, MS polymerase chain reaction (PCR), MLPA, short tandem repeat (STR) linkage analysis, microsatellite analysis, and Southern blotting. Among these, MS-PCR and MLPA remain the gold standard because they are highly sensitive, specific, and efficient in detecting paternal microdeletions of 15q11-15q13 or maternal UPD of chromosome 15 [1]. Maternal UPD 15, responsible for 20-30% of PWS cases, is more common than paternal UPD and has been linked to advanced maternal age. Additionally, trisomy related to Robertsonian translocations may revert to disomy through chromosome loss, leading to UPD in approximately 50% of cases [3]. Clinically, individuals with UPD are less likely to display the typical craniofacial features of PWS than those with deletions [3].

Genotype-phenotype correlations have been described in individuals with PWS. Patients with chromosomal deletions more frequently present with hypopigmentation of the skin, hair, and eyes due to loss of expression of the non-imprinted P gene, which is implicated in oculocutaneous albinism. In contrast, individuals with UPD often demonstrate slightly higher verbal IQ and milder behavioral disturbances. However, psychiatric comorbidities, including psychosis and autism spectrum disorders, are more commonly observed in adults with UPD compared with those with deletions [3].

Endocrine and growth abnormalities are central features of PWS. Short stature is a hallmark of PWS, with absent pubertal growth acceleration and mean untreated heights of 148 cm in females and 155 cm in males [5]. Furthermore, infants with PWS typically exhibit birth weight, length, and body mass index (BMI) values that are approximately 15-20% lower than those of their unaffected siblings, although these parameters often remain within normal limits [6]. Almost all individuals with PWS present with hypogonadism and GH deficiency, while central adrenal insufficiency is uncommon. In contrast, central hypothyroidism is reported in up to 30% of affected children. Although obesity is uncommon during early childhood, it becomes highly prevalent in adulthood (>90%), with type 2 diabetes occurring in up to one-quarter of obese adults [2]. GH deficiency, affecting 40-100% of cases, is considered the most consistent endocrinopathy, reflecting dysfunction of the GH/insulin-like growth factor (GH/IGF-I) axis [7]. A study published in 2022 evaluating GH in a patient with PWS reported that GH therapy was initiated at age 10, with good tolerance and improved growth. After five years, the patient developed obesity (BMI 30.8 kg/m²) and type 2 diabetes, prompting GH discontinuation and antidiabetic treatment. Subsequently, weight loss and glycemic improvement allowed GH reinitiation two years later [2]. In contrast to earlier descriptions of PWS that proposed only two nutritional stages, failure to thrive followed by hyperphagia leading to obesity, recent studies have identified a more complex pattern involving seven distinct phases. Phase 0 occurs in utero, with reduced fetal movement and growth restriction. Phase 1 includes a hypotonic, non-obese infant, with subphase 1a showing feeding difficulties (birth-15 months) and subphase 1b showing steady growth. Phase 2 is marked by weight gain, first without increased appetite (2a, around two years), then with rising food interest (2b, around 4.5 years). Phase 3, the hyperphagic phase, presents with food-seeking and lack of satiety (around eight years). Not all patients experience every phase, though most follow this general progression. Phase 4, observed in some adults, is characterized by reduced appetite and improved satiety [6].

Sleep disorders in PWS are common, heterogeneous in presentation, and frequently underdiagnosed. Excessive daytime sleepiness is a cardinal clinical feature, typically emerging in childhood and often severely impairing daily functioning. Among the various sleep abnormalities, SDB, which includes both obstructive and central sleep apnea, is particularly common in individuals with PWS, with prevalence estimates between 50% and 100%, varying across studies [8]. A case report published in 2014 described a nine-year-old Caucasian girl diagnosed with PWS at 15 months of age. She was born at 41 weeks of gestation, presented with neonatal hypotonia, and required nasogastric tube feeding for one month. Clinical evaluation revealed dysmorphic features suggestive of PWS. The patient was further assessed for abnormal sleep behavior, particularly excessive daytime sleepiness, which is a common feature in PWS. At two years and 10 months, she was referred to a pediatric pulmonologist for polysomnography prior to initiation of GH therapy. Initial overnight oximetry was normal with no evidence of sleep apnea; however, the mother reported recurrent episodes since infancy resembling narcolepsy, characterized by brief collapses with upward eye deviation while maintaining consciousness. These events, often triggered by laughter, occurred three to four times per week and resolved spontaneously. Daytime hypersomnia was also noted, with the child sleeping more than 12 hours at night in addition to prolonged daytime naps. The sleep study demonstrated good overall efficiency but revealed disrupted sleep architecture, with poor cycling through sleep stages and significant fragmentation, particularly during rapid eye movement (REM) sleep [9].

Recurrence risk is largely dependent on the underlying genetic mechanism. Sporadic 15q11.2-15q13 deletions carry a recurrence risk of <1%, whereas paternal balanced rearrangements such as translocations or inversions confer a substantially higher risk, up to 25-50% [3]. Mortality in PWS may occur at any age and is often sudden. In children, respiratory infections are the leading cause of death. In adolescents and young adults, choking episodes, gastrointestinal perforation, and accidental events are more common, while in adulthood, obesity-related complications such as cardiopulmonary failure and thromboembolism predominate [2].

Long-term management requires multidisciplinary follow-up. Current recommendations include annual scoliosis screening beginning in early childhood, ophthalmologic evaluation for strabismus and visual impairment from infancy, and bone density assessment (DEXA) from adolescence due to the elevated risk of osteoporosis [10].

## Conclusions

Accurate and early chromosomal analysis is essential for the diagnosis of PWS and for distinguishing it from other syndromes with overlapping features. Timely identification facilitates the implementation of optimized management strategies, thereby improving long-term outcomes and overall quality of life. While this case highlights the potential benefits of GH, the findings are limited by a short follow-up period. Furthermore, continued research is warranted to refine and standardize clinical practice guidelines, with particular emphasis on early intervention and multidisciplinary follow-up to enhance patient-centered care and long-term prognosis.
